# Concomitant *in situ* and in transit right heart thrombi: a case report

**DOI:** 10.11604/pamj.2020.37.355.27416

**Published:** 2020-12-18

**Authors:** Najlaa Belharty, Rania El Azouzi, Yassmine Chafai, Najat Mouine, Aatif Benyass

**Affiliations:** 1 Department of Cardiology, Mohamed V Military Hospital, Mohammed V University, Rabat, Morocco

**Keywords:** Multiple thrombi, right cardiac chambers, thrombus in transit, *in situ* thrombosis, case report

## Abstract

Right heart thrombi can form in situ or lodge in the right cardiac chambers, originating from deep venous thrombosis. The latter carries a poor prognosis, taking into account the very high mortality rate. We herein report a case of an 83-year-old man who developed thrombus in the inferior vena cava that extended up to the right atrium, along with two distinct masses attached to the right ventricle wall.

## Introduction

In absence of atrial fibrillation, right heart thrombi (RHT) mostly represent an embolus in transit originating from a deep venous thrombosis [1] which has the propensity of distal embolization. They are uncommon but are associated with higher mortality (80-100%) [2]. Right atrial clots may also form *in situ* and are mostly small and immobile. There is little if any consensus for the optimal management of RHT [2], thus being challenging, most importantly, when treating a combination of in transit and *in situ* RHT.

## Patient and observation

An 83-year-old man with a medical history of diabetes mellitus, overt cardiovascular disease, a 10 months history of stage III chronic kidney disease and haematuria was referred to our hospital for dyspnoea, atypical chest pain and persistent light-headedness. The patient experienced a syncopal episode after a strenuous effort. Physical examination revealed a stable hemodynamic status, a pulsus parvus and an increased intensity of pulmonic component of the second heart sound. Electrocardiography revealed no abnormalities except for a low QRS voltage in limb leads.

**Figure 1:**
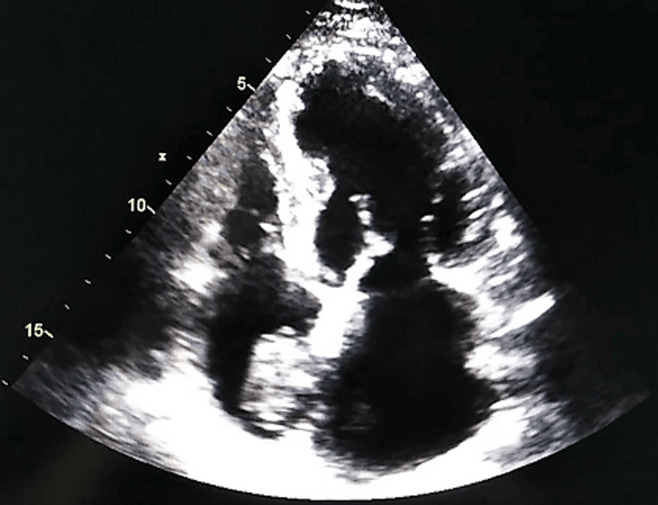
TTE apical four chambers view showing a thrombus in the right atrium, attached to the inter-atrial septum

**Figure 2:**
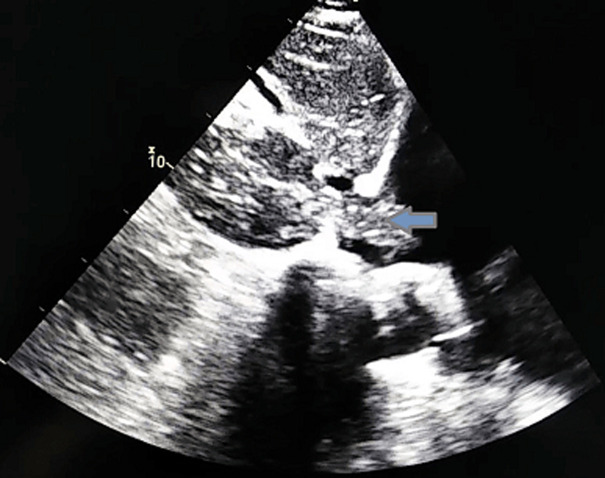
a thrombus originating from the inferior vena cava and protruding into the right atrium on a TTE subcostal view

Laboratory findings highlighted a normocytic normochromic regenerative anaemia, thrombocytopenia and noted high levels of CA19-9. The transthoracic echocardiography (TTE) readily showed two distinct masses occupying the right ventricle outflow tract measuring respectively 15 x 9 mm and 14 x 7 mm, and a large well-defined mass (22 x 33mm) in the right atrium attached to the inter atrial septum (Figure 1), a subcostal view revealed that the mass seemed to originate from the inferior vena cava (IVC) (Figure 2), hence suggestive of a thrombus.

**Figure 3:**
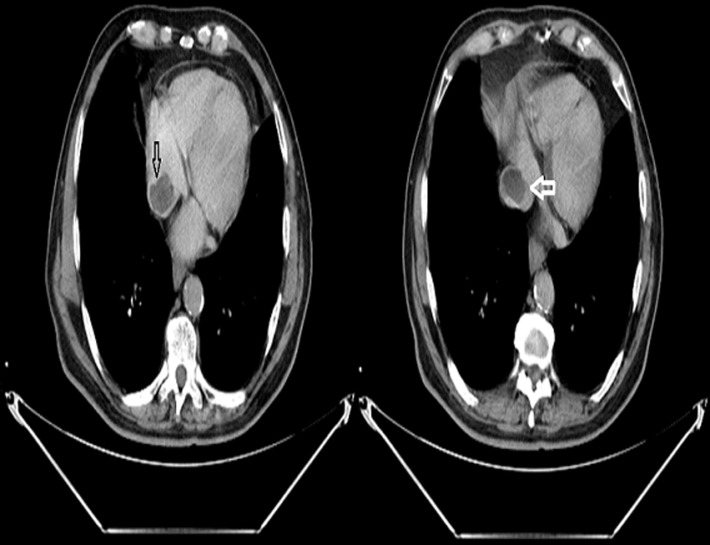
filling defect extending from the inferior vena cava (white arrow) to the right atrium (black arrow)

Computed tomography scan of the chest and abdomen were undergone to rule out any primary malignancy. It consistently showed that the mass is extending from the IVC to the right atrium (Figure 3), along with another mass situated in the right ventricle, without evidence of pulmonary embolism. Moreover, we noted a dilation of the right urinary tract associated to ureteral wall thickening highly suggestive of a tumour, considering the history of haematuria and the presence of malignant cells in urine samples.

In light of this, anticoagulation with intravenous unfractionated heparin was initiated with echocardiographic follow-up. The patient didn´t experience haematuria or any external bleeding in the following days. Yet, after six days of heparin therapy, the patient presented acute respiratory failure and hemodynamic instability refractory to intensive medical care. The patient died shortly after.

## Discussion

Intra-cardiac thrombus can be a complication of various conditions, such as atrial fibrillation, lead-line associated thrombi, advanced heart failure, acute myocardial infarction or venous thromboembolism with a thrombus in transit [3]. Right heart thrombi (RHT) in transit are referred to deep venous thrombi that have mobilized and become lodged in the right heart en route to the pulmonary arteries that may potentially migrate and lead to additional embolic complications. In-hospital mortality has been reported to be as high as 45%, thus, right heart thrombi in transit carry a poor prognosis [4]. TTE is the preferred diagnostic method and helps distinguish between clots formed *in situ* and clots in transit. Transesophageal echocardiography (TEE) provides better visualization of the thrombus and should be considered when TTE is suboptimal or non-diagnostic [1].

Three major types of right heart thrombus can be distinguished on echocardiography: type A, the commonest, is usually the result of deep venous thrombosis and has the highest risk of embolization. It has a worm-like appearance and is freely mobile within the heart chambers. Type B thrombus is thought to originate within the atrium or ventricle; it is firmly attached to the chamber wall and has an ovoid shape. Type C thrombi are rare, highly mobile and resemble cardiac myxomas [1]. Interestingly, the patient in our case had a combination of two types of RHT, type A and B, rendering its management more challenging. Three different therapies have been described: heparin, thrombolytics and surgical or percutaneous catheter removal of RHT [5].

To date, most published reports on RHT are uncontrolled retrospective case series or individual case reports. Furthermore, patients treated with anticoagulation, thrombolytics or surgical embolectomy differ in their baseline characteristics or severity of illness, making direct comparisons between the therapeutic strategies difficult [2]. Thrombolytics have been shown to dissolve right heart thrombi in transit and improve right heart strain, pulmonary vascular resistance and pulmonary hemodynamics. The major risk associated with thrombolytic therapy is significant bleeding and it should hence be avoided in patients with contraindications, including recent surgery or stroke. Surgical embolectomy is considered a classic treatment for RHT in transit and may be the treatment of choice when thrombolytic therapy is contra-indicated. There have been case reports documenting the use of percutaneous catheterization to retrieve right heart thrombi in transit.

Treatment with surgery or thrombolytics has been shown to significantly reduce mortality when compared to treatment with anticoagulation alone [4]. Due to the high complexity of our case, thrombolysis was declined, given the high risk of bleeding of the patient and neither surgical nor percutaneous catheter removal were proposed taking into account the surgical risk and the concomitant presence of *in situ* and in transit intra-cardiac thrombi. The management of this subset of patients remains difficult, considering the scarcity of data on this subject. The initiation of the right heart thrombi European registry (RiHTER) is currently underway and will hopefully provide prospective data on the management of this group of patients [2].

## Conclusion

RHT, whether formed *in situ*, being in transit or existing concomitantly, are rare, yet a highly concerning medical condition. Optimal treatment is still undetermined, but based on the available data, it is clear that, anticoagulation alone is not sufficient. Albeit Thrombolytic agents and surgery have better outcomes, each strategy is considered based on a variety of factors, hemodynamic instability, hemorrhagic risk and availability of surgical embolectomy.
